# Long-term reduced functional capacity and quality of life in hospitalized COVID-19 patients

**DOI:** 10.3389/fmed.2023.1289454

**Published:** 2024-03-06

**Authors:** Anderson Donelli da Silveira, Fernando Luis Scolari, Marina Petersen Saadi, Darshan H. Brahmbhatt, Mauricio Milani, Juliana Goulart Prata Oliveira Milani, Gerson Cipriano Junior, Ivaine Tais Sauthier Sartor, Gabriela Oliveira Zavaglia, Maiko Luis Tonini, Marcela Santos Correa da Costa, Marcelo Comerlato Scotta, Renato T. Stein, Regis Goulart Rosa

**Affiliations:** ^1^Division of Cardiology, Hospital de Clínicas de Porto Alegre, Porto Alegre, Brazil; ^2^Postgraduate Program in Cardiology, Universidade Federal do Rio Grande do Sul, Rio de Janeiro, Brazil; ^3^Ted Rogers Centre for Heart Research, Peter Munk Cardiac Centre, University of Toronto, Toronto, ON, Canada; ^4^Social Responsibility, Hospital Moinhos de Vento, Porto Alegre, Brazil; ^5^Hasselt University, REVAL/BIOMED, Hasselt, Belgium; ^6^Health Sciences and Technologies Graduate Program, University of Brasilia (UnB), Brasilia, Brazil; ^7^General Coordination, Chronic and Airborne Disease Surveillance Coordination, Health Surveillance Secretariat, Brazilian Ministry of Health, Brasilia, Brazil; ^8^General Coordination, National Immunization Program, Health Surveillance Secretariat, Brazilian Ministry of Health, Brasilia, Brazil

**Keywords:** CPET cardiopulmonary exercise testing, COVID-19, long COVID, HRQOL, functional capacity

## Abstract

**Background:**

Persistent symptoms and exercise intolerance have been reported after COVID-19, even months after the acute disease. Although, the long-term impact on exercise capacity and health-related quality of life (HRQoL) is still unclear.

**Research question:**

To assess the long-term functional capacity and HRQoL in patients hospitalized due to COVID-19.

**Study design and methods:**

This is a prospective cohort study, conducted at two centers in Brazil, that included post-discharge COVID-19 patients and paired controls. The cohort was paired by age, sex, body mass index and comorbidities, using propensity score matching in a 1:3 ratio. Patients were eligible if signs or symptoms suggestive of COVID-19 and pulmonary involvement on chest computed tomography. All patients underwent cardiopulmonary exercise testing (CPET) and a HRQoL questionnaire (SF-36) 6 months after the COVID-19. The main outcome was the percentage of predicted peak oxygen consumption (ppVO2). Secondary outcomes included other CPET measures and HRQoL.

**Results:**

The study sample comprised 47 post-discharge COVID-19 patients and 141 healthy controls. The mean age of COVID-19 patients was 54 ± 14 years, with 19 (40%) females, and a mean body mass index of 31 kg/m^2^ (SD, 6). The median follow-up was 7 months (IQR, 6.5–8.0) after hospital discharge. PpVO2 in COVID-19 patients was lower than in controls (83% vs. 95%, *p* = 0.002) with an effect size of 0.38 ([95%CI], 0.04–0.70). Mean peak VO2 (22 vs. 25 mL/kg/min, *p* = 0.04) and OUES (2,122 vs. 2,380, *p* = 0.027) were also reduced in the COVID-19 patients in comparison to controls. Dysfunctional breathing (DB) was present in 51%. HRQoL was significantly reduced in post COVID patients and positively correlated to peak exercise capacity.

**Interpretation:**

Hospitalized COVID-19 patients presented, 7 months after discharge, with a reduction in functional capacity and HRQoL when compared to historical controls. HRQoL were reduced and correlated with the reduced peak VO2 in our population.

## Introduction

The COVID-19 pandemic declared in March of 2020 resulted in a massive number of cases in several countries (1). SARS-Cov-2 infection overloaded healthcare systems and was responsible for over 450 million cases worldwide (2). Viral pneumonia is the hallmark of hospitalized COVID-19 patients, and, in severe forms, progress to acute respiratory distress syndrome (ARDS), the most worrying presentation with a high mortality rate and associated with long-term disabilities (3).

Experience from the previous severe acute respiratory syndrome (SARS-CoV-1) epidemic, suggests that pulmonary function at rest and exercise capacity could be profoundly impaired, either by the virus action or because of post-intensive care syndrome, but its long-term impact is unknown (4–6). Studies conducted in patients who recovered from COVID-19 have related a myriad of symptoms, including chest pain, fatigue, dyspnea, leg pain and weakness (7, 8). A case-control study conducted at 2–3 months from disease onset showed that a significant proportion of hospital discharged patients reported symptoms such as breathlessness, fatigue, depression and limited exercise capacity (9). Furthermore, cross-sectional studies performing cardiopulmonary exercise testing (CPET), the gold-standard for functional capacity assessment, elucidated some exercise limitation pathophysiological mechanisms (10, 11). Studies conducted 3 months after discharge had shown reduced functional capacity in 33 to 50% of patients post COVID-19 (12, 13). However, these studies only evaluated short-term physical impairment after COVID-19 infection, with uncertainty about causality, mechanisms of limitation and persistence of this limitation. Possible underlying mechanisms for these persistent complaints can include cardiac, pulmonary and peripheral (oxygen extraction) limitations, with either two or more combined.

The impact in health-related quality of life (HRQol) have been shown to be impaired in patients post COVID-19 (14). Countless patients affected by COVID-19 are returning to their work activities, and the real burden of this disease is still being discovered. Therefore, the aim of this study was to assess long-term functional capacity and HRQoL, among survivors of hospitalization due to COVD-19, comparing the results with those of historical controls matched by age, sex, body mass index, and comorbidities.

## Methods

This is a prospective cohort study of COVID-19 patients who required hospitalization due to respiratory symptoms between June 2020 and December 2020 and paired historical controls. Participants were recruited from a previous cohort, in which adult patients (≥18 years) were eligible if admitted with signs or symptoms suggestive of COVID-19 (cough, fever, or sore throat) within 14 days of onset and hospitalized in the prior 2 days (15). All patients were hospitalized at a private hospital in Porto Alegre, southern Brazil. This private institution is the reference hospital in the care of COVID-19 cases in Porto Alegre, RS, Brazil, with 372 infirmary beds, and 113 ICU beds.

Between six and nine months after hospital discharge, patients with confirmed COVID-19 by RT-PCR and pulmonary involvement on chest computed tomography were contacted by telephone to perform a CPET and a clinical evaluation through a HRQoL questionnaire. A physician assessed the presence of persistent symptoms during the clinical evaluation. Exclusion criteria were inability to perform CPET due to musculoskeletal limitation, absence of radiologic pulmonary involvement and patient refusal. The project was submitted to the local ethics committee and complied with both the National Health Council Resolution 466/12 and the Declaration of Helsinki. All patients signed an informed consent.

### Data collection

All data were collected prospectively including demographic, symptoms at admission, comorbidities, need for oxygen support, supplemental ventilatory support type, need for intensive care and length of stay. Oxygen support therapy was defined as the therapy used with the highest oxygen concentration supply and invasiveness during hospital admission. Patients were also classified according to the World Heart Organization COVID-19 severity classification: mild (symptomatic patients meeting the case definition for COVID-19 without evidence of viral pneumonia or hypoxia); moderate (adults with clinical signs of pneumonia (fever, cough, dyspnea, fast breathing) but no signs of severe pneumonia, including SpO2 ≥ 90% on room air); severe (adults with clinical signs of pneumonia plus one of the following: respiratory rate > 30 breaths/min; severe respiratory distress; or SpO2 < 90% on room air); and critical patients with acute respiratory distress syndrome (ARDS) or sepsis or septic shock (16). At the follow-up visit, patients were interviewed to assess persistent symptoms, medications in use, current exercise activity and other clinically relevant information.

### Cardiopulmonary exercise testing

CPET was performed on a treadmill (General Electric T-2100, GE Healthcare, United States) with breath-by-breath gas analysis (Metalyzer 3B, Cortex, Leipzig, Germany) between January 2021 to March 2021. Symptom-limited maximal exercise testing with an individualized ramp protocol was used to yield fatigue-limited exercise duration of 8 to 12 min. Peak VO2 was determined by the higher measure of 20 s averaging of breath-by-breath values. Other prognostic variables were also measured, such as first and second ventilatory thresholds, which were defined by V-slope for first ventilatory threshold and ventilatory equivalent method to confirm first and determine second ventilatory threshold, minute ventilation-carbon dioxide output relationship (VE/VCO2 slope), oxygen uptake efficiency slope (OUES) and resting end tidal carbon dioxide tension. Maximal effort was considered when respiratory exchange ratio (RER) was equal to or above 1.05. Before each test, brief spirometry was performed before each test, to assess forced vital capacity (FVC) and forced expiratory volume in the 1st second (FEV1). Maximal voluntary ventilation (MVV) was estimated by FEV1x 37.5 (17). Peak VE was also compared as a percentage of maximal predicted using a validated equation (18). For the percentage predicted peak VO2 (ppVO2) both the Wasserman’s and Hansen algorithm and FRIEND equations were used (19). Dysfunctional breathing (DB) was defined by pattern recognition as described by previous studies (20, 21). For this classification, we considered the graphs of minute ventilation (VE) versus time, VE/VCO2 slope and respiratory rate (breaths per minute), tidal volume (mL/min) vs. VE (L/min). CPET and spirometry were performed following current guidelines for exercise testing (22).

### Quality of life assessment

Short Form36 (SF-36) physical and mental health questionnaire was completed by all post COVID-19 patients. The SF-36 addresses HRQoL in eight domains (general health, physical functioning, physical role function, bodily pain, vitality, emotional role function, mental health, and social functioning) that are summarized in two dimensions: physical and mental. Scores range from worst to best (0–100). The eight different scales scores were calculated and computed. For construction of summary measures, scales were standardized using a *Z*-score transformation, providing both physical and mental composite scores (PCS and MCS). We used national normative data for both z-scores calculations and for comparison purpose with our sample (23).

### Selection of healthy controls and pairing

Control subjects were selected from a CPET database of 4,957 test subjects without diagnosed cardiovascular or pulmonary disease, evaluated at an experienced laboratory in the Brazilian Midwest region from 2011 to 2020. CPET were mainly performed for cardiorespiratory fitness assessment and exercise prescription. Test subjects who did not fulfill ventilatory maximality criterion (RER ≥1.05) were excluded before pairing. COVID-19 patients were matched with controls by a 1:3 ratio for age, sex, BMI, hypertension and diabetes. A nearest neighbor matching method was applied with a caliper of 0.2 without replacement. After matching, included variables were compared between groups to confirm that there were no significant differences.

### Statistical analysis

Continuous data were tested for normality with Shapiro–Wilk test and presented as mean (standard deviation) or median (interquartile range). Categorical data are presented as absolute count and relative frequency. Comparisons between COVID-19 and matched controls were performed by independent samples Student’s *t*-test and chi-square test. The effect size was calculated by dividing the mean difference between groups by the standard deviation of the population. Spearman’s rank correlation coefficient was performed to test association of HRQoL and CPET data. Non-linear regression with curve fitting was used to examine the relationship between peak VO_2_ and PCS of HRQol. We used a generalized linear model to estimate the association of COVID-19 infection in comparison to healthy controls for the ppVO2. An adjusted model including age, sex, height, and weight was also performed. This study used a convenience cohort of patients. We performed a post-hoc power calculation for the observed differences of the ppVO2 among COVID-19 and healthy controls resulting in a power of 89.94% for an alpha value of 5%. Significance was accepted at *p* < 0.05 for all tests. Data were analyzed in SPSS, Version 25.0 for Windows (SPSS Inc., Chicago, IL, United States) and R 4.1.1 statistical software (R: The R Project for Statistical Computing, https://www.r-project.org).

## Results

**Figure 1:**
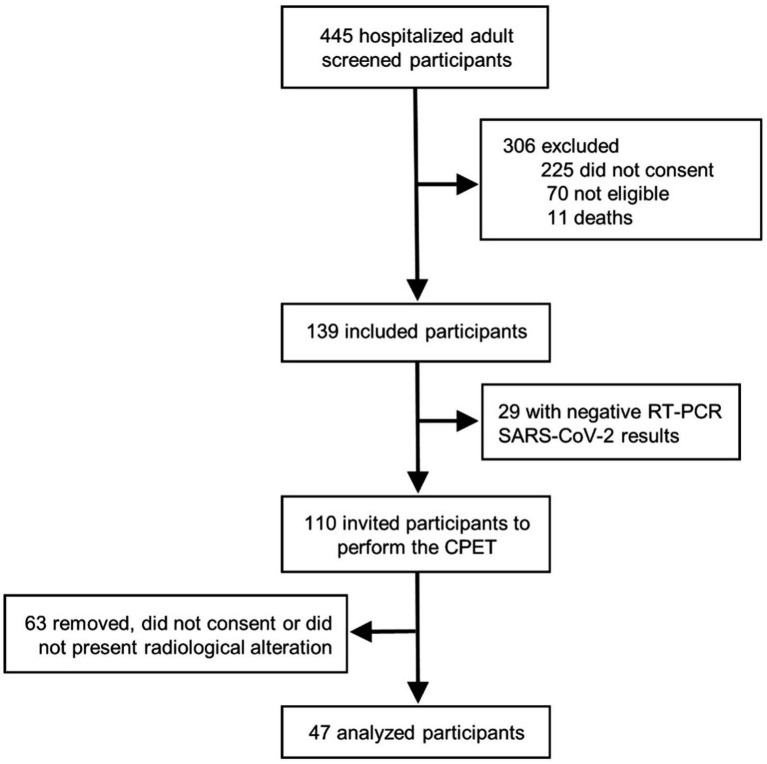
Study design. We screened 445 hospitalized patients diagnosed with COVID-19 infection and selected for eligibility when a positive polymerase chain reaction test and signs of lung involvement evaluated by computed tomography chest imaging. Of the 110 eligible patients, 47 accepted the invitation to perform a cardiopulmonary exercise test study 6 months after hospital discharge.

From 110 screened patients with confirmed SARS-CoV-2 infection, 63 were excluded due to an absence of radiological abnormalities at time of admission or did not consent to perform CPET. The flowchart of the study is shown in Figure 1. Our sample comprised 47 previously hospitalized COVID-19 patients with a mean age of 54 years (standard deviation [SD], 14), 19 (40%) females, 24 (51%) with hypertension and 12 (26%) with diabetes. There were no significant differences found in baseline characteristics between cases and the 141 matched controls. COVID-19 patients required supplementary oxygen in 26 (55%) cases, but only 3 (6%) required high-flow nasal cannula, 1 (2%) Bilevel, and 2 (4%) mechanical ventilation. Patients were hospitalized for a median of 7 days (interquartile range [IQR], 4–10) and 5 (11%) required ICU admission. Twenty-six (55%) subjects reported persistent symptoms, being fatigue (46%), dyspnea (38%), and leg pain/weakness (21%) the most common. Table 1 summarizes demographic and clinical data from COVID-19 patients and healthy controls.

**Table 1:** Demographic and clinical characteristics of COVID-19 patients and healthy controls.

	Control subjects (*N* = 141)	COVID-19patients (*N* = 47)	*p*
Age, years	54 ± 12	54 ± 14	0.97
Female sex, %	57 (40%)	19 (40%)	1.00
BMI, kg/m^2^	31 ± 5	31 ± 6	0.83
Hypertension, %	60 (43%)	24 (51%)	0.36
Diabetes, %	36 (26%)	12 (26%)	1.00
Coronary artery disease, %	–	2 (4%)	
Ventilatory support			
Supplementary oxygen, %		26 (55%)	
High-flow nasal cannula, %		3 (6%)	
Non-invasive mechanical ventilation %		1 (2%)	
Invasive mechanical ventilation, %		2 (4%)	
Vasopressor, %		5 (11%)	
ICU admission, %		5 (11%)	
Hospital LOS, days (median, IQR)		7 (4–10)	
Hemoglobin, g/dl (mean ± SD)		13.7 ± 2	
Hematocrit, % (mean ± SD)		40 ± 4	
Leukocyte count × 10^3^ (mean ± SD)		5.4 (3.8–6.9)	
Creatinine, mg/dL (mean ± SD)		0.86 (0.78–1.09)	
D-dimer × 10 2, ng/mL (median, IQR)		635 (391–917)	
US-troponine, (ng/dL) (median, IQR)		0.6 (0.5–0.9)	
WHO COVID-19 severity classification			
-Moderate		33 (70%)	
-Severe/critical		14 (30%)	

BMI, body mass index; CAD, coronary artery disease, CPAP, continuous positive airway pressure; ICU, intensive care unit; LOS, length of stay; US, ultra-sensible; WHO, world health organization.

### Exercise capacity and CPET results

**Figure 2:**
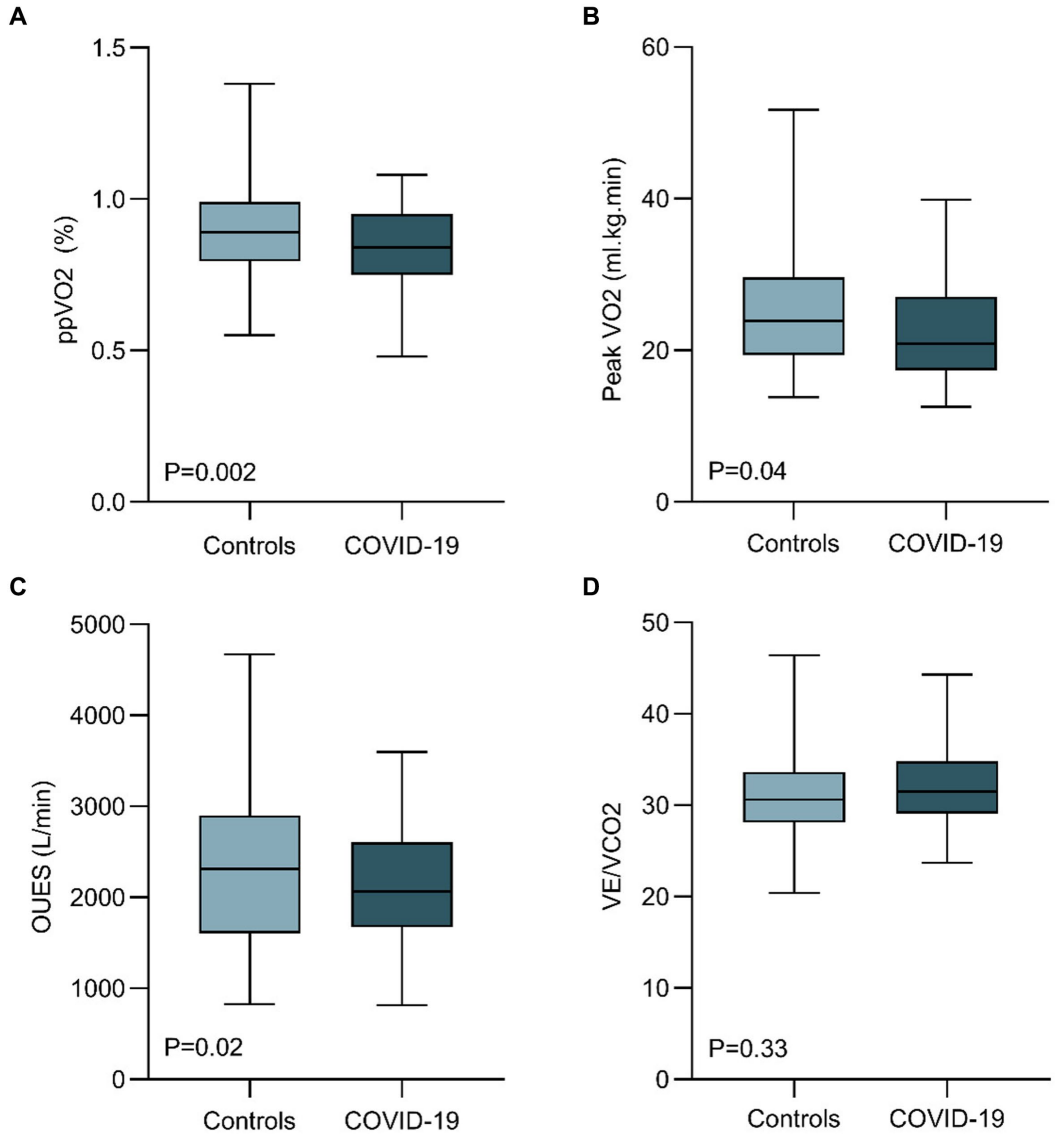
Comparison of cardiopulmonary exercise test parameters between healthy controls and hospitalized COVID-19 patients 6 months after discharge. COVID-19 patients 6 months after hospital discharge showed a reduced ppVO2 (calculated by the Wasserman and Hansen Algorithm), peak VO2, and OUES. VE/VCO2 were similar between cohorts. (OUES, oxygen uptake efficiency slope; ppVO2, predicted-percentage peak VO2; VE, ventilation).

Table 2 and Figure 2 summarize the CPET variables compared between COVID-19 and controls. The median time from hospital discharge to CPET was 7 months (IQR, 6.5–8). The mean FEV1 was 3.34 L [SD, 0.7], and the mean FVC was 4.3 L [SD, 0.9]. Among the patients, 1 presented with a restrictive pattern, while 2 exhibited an obstructive pattern during spirometry. COVID-19 patients showed lower mean ppVO2 by the Wasserman and Hansen algorithm (83% [SD, 15] vs. 95% [SD, 35]; *p* = 0.002), and by the FRIEND equation (81% [SD, 18] vs. 88% [SD, 18]; *p* = 0.039). Peak VO2 (22.5 [SD, 6] vs. 25.0 [SD, 7] mL/kg/min; *p* = 0.048), VO2@AT (14.4 [SD, 4] vs. 12.8 [SD, 3] mL/kg/min; *p* = 0.007), and OUES (2,122 [SD, 611] vs. 2,380 [SD, 860]; *p* = 0.02) were also impaired in COVID-19 patients when compared to healthy controls. The greatest effect size was observed for VO2@AT, ppVO2, peak VO2 and OUES, respectively. Peak VE and percent-predicted VE showed lower values for COVID-19 patients (76 [SD, 23] vs. 84 [SD, 27], *p* = 0.08 and 103 [SD, 40] vs. 93 [SD, 39]; *p* = 0.116, respectively), but without statistical significance. Breathing reserve was normal in all post-COVID patients, with the average peak VE/MVV relation of 0.63 (SD, 0.2).

**Table 2:** Comparison of cardiopulmonary exercise testing between healthy controls and hospitalized COVID-19 patients.

	Control subjects (*N* = 141)	COVID-19patients (*N* = 47)	Effect size	*p*
ppVO2 (%)*	95 ± 35	83 ± 15	0.38 (0.05–0.71)	0.002
ppVO2 (%)**	88 ± 18	81 ± 18	0.39 (0.05–0.72)	0.039
Peak VO2 (mL/kg/min)	25.0 ± 7	22.5 ± 6	0.37 (0.04–0.70)	0.04
OUES	2,380 ± 860	2,122 ± 611	0.32 (0.01–0.65)	0.02
VO2@AT (mL/kg/min)	14.4 ± 4.4	12.8 ± 3.1	0.39 (0.05–0.72)	0.007
Peak oxygen pulse (mL/beat)	13.8 ± 3.9	13.2 ± 3.6		0.15
Peak VO2 (l/min)	2.20 ± 0.8	2.02 ± 0.7		0.19
Peak HR (bpm)	156 ± 22	156 ± 18		0.87
Peak SBP (mmHg)	169 ± 26	164 ± 20		0.23
Peak RER	1.17 ± 0.1	1.18 ± 0.1		0.47
VE/VCO2 slope	31 ± 8	31 ± 7		0.33
Peak VE (l/min)	84 ± 27	76 ± 23		0.08
ppVE (%)	106 ± 40	93 ± 39		0.12

ppVO2, predicted-percentage peak VO2; HR, heart rate; OUES, oxygen uptake efficiency slope; VO2@AT, VO2 at anaerobic threshold; SBP, systolic blood pressure; RER, respiratory exchange ratio; VE, ventilation, ppVE predicted-percentage peak VE. *Wasserman and Hansen algorithm and **FRIEND equation. Continuous data are presented as mean ± standard deviation or median (p25–p75).

When considering those participants with ppVO2 less than 80% of predicted, 21 (45%) COVID-19 patients had values below this threshold, in comparison to only 12 (8.5%) of healthy controls (*p* = 0.01). When using ERS algorithm (24) for determining causes of exercise limitation of these 21 COVID-19 patients, 12 (57%) showed findings consistent with cardiocirculatory limitation and nine subjects (43%), findings suggesting peripheral muscle limitation. It is noteworthy that none of the patients showed reduced breathing reserve or signs of pulmonary limitation.

**Figure 3:**
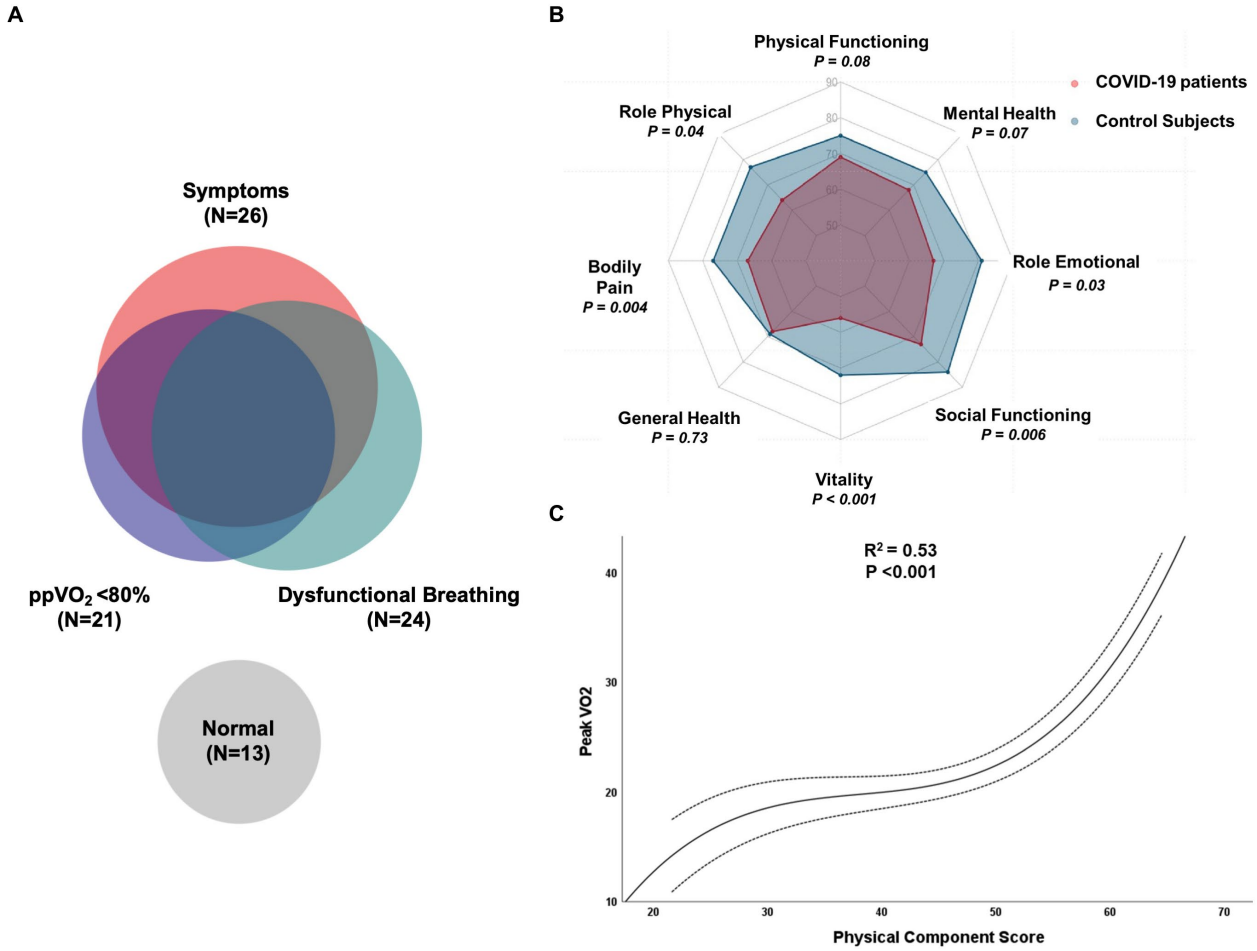
**(A)** Venn diagram illustrating the relationship between symptoms, reduced percent-predicted peak oxygen consumption, dysfunctional breathing and normal evaluation in COVID-19 patients. **(B)** Evaluation of quality-of-life domains of SF-36 between healthy controls and hospitalized COVID-19 patients six months after discharge; **(C)** Cubic regression between peak oxygen consumption during CPET and physical component score of HRQol in COVID-19 patients.

Dysfunctional breathing was prevalent among COVID-19 patients (51%). Persistence of symptoms (dyspnea, fatigue, leg weakness) was associated with the DB ventilatory pattern (OR, 3.8; 95% CI, 1.3–12.1). DB was more common in patients who had lower ppVO2 (78% vs. 89%, *p* = 0.012) and among those with peripheral muscle limitation than cardiocirculatory or normal findings (89% vs. 66% vs.31%, respectively, *p* = 0.005). The relationship between symptoms, DB and reduced ppVO2 is displayed in Figure 3A.

### Predictors of decrease predicted peak VO2

We subsequently performed an analysis to evaluate the impact of COVID-19 in the observed ppVO2 (by Wasserman and Hansen algorithm) in comparison to matched controls (Table 3). COVID-19 patients had a reduced ppVO2 with an unadjusted odds ratio (OR) of 0.89 (95%CI, 0.82–0.95; *p* = 0.002) and an adjusted OR of 0.88 (95%CI, 0.82–0.95, p = 0.002). We then sought to evaluate which characteristics were associated with the ppVO2 in hospitalized COVID-19 patients after discharge (Table 3). The presence of coronary artery disease (OR, 0.83; 95%CI, 0.76–0.89; *p* < 0.001), the use of Bilevel (OR, 0.83; 95%CI, 0.79–0.88; *p* < 0.001), mechanical ventilation (OR, 0.88; 95%CI, 0.79–0.98; *p* = 0.02), and in-hospital length of stay (OR, 0.99; 95%CI, 0.99–0.99; *p* = 0.001) were associated with lower ppVO2.

**Table 3:** Crude and adjusted analysis of variables related to percent-predicted peak VO2 after COVID-19 hospitalization.

	ppVO2 (%)		
	OR	95% CI	*p* value
COVID-19 vs Controls			
Unadjusted	0.92	0.88–0.97	0.003
Adjusted^a^	0.92	0.87–0.97	0.002
COVID-19			
Age (years)	0.99	0.99–1.00	0.09
Male sex	0.94	0.87–1.02	0.15
BMI (kg/m^2^)	1.00	0.99–1.01	0.10
Hypertension	0.95	0.87–1.03	0.25
Diabetes	0.96	0.87–1.06	0.49
CAD	0.83	0.76–0.89	<0.001
Advanced oxygen support	0.91	0.79–1.04	0.20
-High flow cannula	0.94	0.76–1.16	0.57
-Bilevel	0.83	0.78–0.88	<0.001
-Mechanical ventilation	0.88	0.79–0.98	0.02
ICU admission	0.94	0.81–1.09	0.45
Hospital length of stay (days)	0.99	0.99–0.99	0.001
WHO COVID-19 Severe/Critical	0.89	0.80–1.00	0.06

Bilevel, bi-level positive airway pressure; BMI, body mass index; CAD, coronary artery disease; CI, confidence interval; CPAP, continuous positive airway pressure; CPET, cardiopulmonary exercise testing; ICU, intensive care unit; OR, odds ratio; ppVO2, predicted-percentage peak VO2; WHO, world health organization. Continuous data are presented as mean ± standard deviation or median (p25–p75).

a Adjusted for age, sex, height, and weight.

### Quality of life assessment

Quality of life measurements were compared to national normative data stratified by age and sex. Both physical (45 vs. 49; *p* = 0.01) and mental (47 vs. 51; *p* = 0.04) composite mean scores were significantly reduced in COVID-19 patients. Regarding the domains of physical and mental health, vitality (55 vs. 71; p < 0.001), bodily pain (66 vs. 77, *p* = 0.004), role physical (64 vs. 76, *p* = 0.04), role emotional (68 vs. 81, *p* = 0.03) and social functioning (72 vs. 84, *p* = 0.006) were significantly reduced in post COVID-19 subjects. Physical functioning (69 vs. 75, *p* = 0.08) and mental health (69 vs. 74, *p* = 0.07) also were reduced in post-COVID-19 patients, but without statistical significancy. Interestingly, the global health perception of patients was not reduced when compared to controls (68 vs. 69, *p* = 0.73). HRQol results are summarized in Figure 3B.

Physical composite score (Spearman’s *ρ* = 0.654; *p* < 0.001), functional capacity (*ρ* = 0.649; *p* < 0.001) and bodily pain (*ρ* = 0.637; *p* < 0.001) showed a significant, moderate correlation with peak VO2 in our sample. The relationship between peak VO2 and PCS was best described as a third-degree polynomial, presenting a moderate coefficient of determination (*R*^2^ = 0.53; *p* < 0.001) as shown in Figure 3C.

## Discussion

Our study shows that hospitalized COVID-19 patients, even after more than 6 months post-discharge, can still demonstrate reduced functional capacity and HRQoL compared to matched controls. Several CPET prognostic markers, physical and mental aspects of HRQoL were also significantly reduced 6 months after hospital discharge in COVID-19 patients, demonstrating the long-term impact of the disease. Moreover, more than half of the patients has persistent symptoms at 6 months follow-up, increasing the burden of disease.

Our results are consistent with those found in previous studies evaluating patients in the short-term after COVID-19 infection (12, 13). Skjorten and colleagues, using a treadmill, found one-third of patients with a ppVO2 less than 80%, additionally, 15% percent of these patients had shown reduced ventilatory efficiency (12). Clavario et al. reported one-third of patients with a reduced peak VO2 3 months post-discharge on cycle ergometer CPET, mostly due to muscular impairment (13). Many recent data suggest that peripheral factors are incriminated in persistent functional impairment in post COVID-19 patients. A small study was conducted in 10 patients without cardiopulmonary disease who recovered from COVID-19. Patients were investigated with invasive cardiopulmonary exercise testing (iCPET) and compared to 10 age- and sex-matched controls (25). The reduction in peak VO2 was associated with impaired systemic oxygen extraction, depicting a peripheral rather than a central cardiac limitation.

Functional impairment after COVID-19 infection remains a major concern. We demonstrated that after 6 months of discharge, COVID-19 patients had a reduction in ppVO2 and peak VO2 when compared to matched controls. The observed higher peak VO2 in males was not confirmed by the ppVO2, suggesting an absence of sex-related post-COVID-19 hospitalization functional impairment. Interestingly, we did not find any exercise limitation due to pulmonary gas exchange or ventilatory mechanics. In keeping with previous reports, cardiocirculatory limitation was the predominant deficit encountered in our study. A recent meta-analysis explored the utility of CPET to evaluate long COVID-19 symptoms in adults, showing that exercise capacity was reduced in these patients and that CPET may provide insight into the mechanisms for this impairment (26).

Several patients after COVID-19 had presented a rapid and irregular breathing pattern consistent with DB, which is characterized sometimes by rapid shallow breaths or other erratic ventilatory patterns (20, 21). It was associated with persistent symptoms such as dyspnea and fatigue, and with a reduced ppVO2 as well. We have found a similar prevalence of DB when comparing our data to other studies, also showing a positive correlation of this ventilatory abnormality with symptoms (20, 27). Nevertheless, identification of DB is subjective and requires pattern recognition, without any strict criteria. The development of quantitative methods would help us to diagnose this entity.

Notably, the requirement of Bilevel support, mechanical ventilation, ICU admission, hospital length of stay, and COVID-19 severity were all associated with a reduced ppVO2. The high number of COVID-19 infected patients will certainly impact the demand for dyspnea evaluation and referrals for rehabilitation soon. We should be aware that symptoms persist even 6 months after hospital discharge in COVID-19 patients. A preemptive approach towards rehabilitation could be beneficial, especially in those more likely to be impacted such as in those with severe disease presentations. Physical rehabilitation after discharge could improve these symptoms, especially in patients with a severe initial COVID-19 presentation, but the efficacy of this intervention is yet to be established in this scenario (27).

Mental and physical aspects of HRQoL were significantly reduced in COVID-19 patients 6 months after discharge. A reduced mental aspect of HRQoL is consistent with the findings of sleep disturbances, depression, anxiety, and cognitive impairment as reported in a systematic review (28). Of note, the comparison of HRQoL scores was adjusted by age and sex according to national normative data, which strengthens the evidence for this impairment when compared to the general population. Both peak VO2 and ppVO2 were positively correlated with several aspects of HRQoL, not only physical, but also social and mental. It provides a better understanding of persistent impairment after moderate to severe COVID-19: there is a pathophysiological basis for these symptoms associated with a documented reduction in exercise capacity.

Our study has several limitations. Although we used a 3:1 control ratio, our study cannot support that the late exercise impairment observed in COVID-19 patients is related exclusively to this etiology. Comparing CPET parameters after hospital discharge with a population affected by another viral pneumonia could better clarify if COVID-19 is responsible for these symptoms or they are merely due to the hospital stay. One of the variables most affected in post COVID subjects is the diffusion capacity, which was not measured in our study. Recruitment to the study is another limitation. The stigma related to COVID-19 infection and the environmental safety for a CPET study were barriers to patient recruitment. Although the selection was not based on the presence of symptoms, patients more likely to present dyspnea or fatigue could be more prone to accept the research invitation. Our inclusion criteria limited the results to hospitalized patients with pulmonary involvement, so caution should be taken in extrapolating these findings to less severe patients.

## Conclusion

Hospitalized COVID-19 patients showed decreased exercise capacity after 6 months from discharge related mainly to cardiocirculatory impairment and peripheral muscle limitation. Dysfunctional breathing was common and associated with persistent symptoms. Both physical and mental quality of life domains were reduced in these patients. The requirement of higher level of oxygen support, intensive care admission, longer hospital stay, and COVID-19 severity were the main predictors of reduced peak VO2. Our results highlight the health support required by these patients even after more than 6 months from hospital discharge.

## Data availability statement

The raw data supporting the conclusions of this article will be made available by the authors, without undue reservation.

## Ethics statement

The studies involving humans were approved by Comitê de Ética do Hospital de Clínicas de Porto Alegre. The studies were conducted in accordance with the local legislation and institutional requirements. The participants provided their written informed consent to participate in this study.

## Author contributions

AS: Writing – original draft, Writing – review & editing. FS: Writing – original draft, Writing – review & editing. MSa: Writing – original draft, Writing – review & editing. DB: Writing – review & editing. MM: Writing – original draft. JM: Writing – review & editing. GJ: Writing – review & editing. IS: Writing – review & editing. GZ: Writing – review & editing. MT: Writing – review & editing. MC: Writing – review & editing. MSc: Writing – original draft. RS: Writing – review & editing. RR: Writing – review & editing.
